# Polypharmacy and Health-Related Quality of Life/Psychological Distress Among Patients With Chronic Disease

**DOI:** 10.5888/pcd19.220062

**Published:** 2022-08-18

**Authors:** Lisa Van Wilder, Brecht Devleesschauwer, Els Clays, Peter Pype, Sophie Vandepitte, Delphine De Smedt

**Affiliations:** 1 Department of Public Health and Primary Care, Ghent University, Ghent, Belgium; 2 Department of Epidemiology and Public Health, Sciensano, Brussels, Belgium; 3 Department of Translational Physiology, Infectiology and Public Health, Ghent University, Merelbeke, Belgium

## Abstract

**Introduction:**

To date, no study has investigated the impact of polypharmacy (use of ≥5 medications concurrently) on health-related quality of life (HRQOL) and psychological distress in a combined sample of chronic disease patients and patients with multimorbidity, using diverse HRQOL measures. This study aimed to explore the association between polypharmacy and HRQOL/psychological distress by using data from a cross-sectional study in Flanders (Belgium).

**Methods:**

We analyzed cross-sectional survey data on 544 chronically ill patients recruited from June 2019 through June 2021. HRQOL was measured with the EuroQol-5 Dimension-5 Level questionnaire (EQ-5D-5L) and the 12-Item Short Form Health Survey (SF-12); psychological distress was measured with the Hospital Anxiety and Depression Scale (HADS). Multiple linear regression models were built to assess the association between polypharmacy and HRQOL/psychological distress.

**Results:**

Overall, compared with patients without polypharmacy, patients with polypharmacy reported worse EQ-5D-5L index values, EuroQol visual analogue scale (EQ-VAS) scores, SF-12 physical component scores (PCS), SF-12 mental component scores (MCS), and HADS anxiety and depression subscales. In the final regression model adjusting for age, sex, educational attainment, and multimorbidity, polypharmacy remained significantly associated with lower HRQOL in terms of the EQ-5D-5L index (β = −0.12; *P* = .008), EQ-VAS (β = −0.11; *P* = .01), and SF-12 PCS (β = −0.15; *P* = .002) but not with psychological distress (HADS) and SF-12 MCS.

**Conclusion:**

This study found that polypharmacy was negatively associated with the physical domain of HRQOL, but not with the mental domain, among patients with chronic diseases. These results may be especially important for patients with multimorbidity, given their greater risk of polypharmacy.

**Summary**

**What is known on this topic?**

Chronically ill patients tend to have a high risk of multimorbidity; hence, multiple drug use is common. The resulting polypharmacy (use of ≥5 medications), however, increases the risk of adverse drug–drug or drug–disease interactions, which may negatively affect patients’ health-related quality of life (HRQOL).

**What is added by this report?**

No significant associations were found for the mental component of HRQOL or psychological distress, suggesting an unfavorable effect of polypharmacy only on the physical domain of patients’ HRQOL.

**What are the implications for public health practice?**

Results support the need for health care professionals to recognize drug-related adverse events and the negative effects of polypharmacy on HRQOL, especially among patients with multimorbidity.

## Introduction

During the past few decades, chronic diseases have predominated over infectious diseases, and their prevalence is still rising (1). In addition, people with chronic conditions often have multimorbidity — multiple chronic conditions at the same time (2). Having 1 or more chronic conditions has negative effects on a person’s well-being and health-related quality of life (HRQOL) (3–5). In addition to optimal longevity, an ultimate goal of modern health care is to achieve the best possible life for patients in terms of an optimal HRQOL, especially since the shift from problem-oriented care to goal-oriented care (6). HRQOL has become an important outcome because it captures a person’s self-perceived physical, mental, and social functioning (6,7).

Because chronically ill patients tend to have a higher risk of multimorbidity, multiple drug use is common (8,9). The resulting polypharmacy, defined as the use of 5 or more medications (10,11), however, increases the risk of adverse drug–drug or drug–disease interactions and the risk of a “prescription cascade” (ie, the process whereby side effects of drugs are misdiagnosed as symptoms of another medical event and lead to an additional prescription [12]), which may negatively affect a patient’s HRQOL (13,14). The association between polypharmacy and low HRQOL has been documented for single health conditions such as end-stage kidney disease (15), arthritis (16), and cardiometabolic risk factors (17). However, to date, no study has investigated the impact of polypharmacy on HRQOL in a combined sample of chronic disease patients and patients with multimorbidity by using diverse HRQOL measures. Hence, this study aimed to explore the association between polypharmacy and HRQOL among people with chronic conditions and multimorbidity by using data from a cross-sectional study in Flanders (Belgium).

## Methods

### Study design

We used data from a cross-sectional study (the QAPICHE study, an acronym for “quality of life in patients with chronic disease”) conducted in Flanders, Belgium. Detailed information on the study aims and methodology can be found elsewhere (18). In brief, the QAPICHE study provides insight into the HRQOL of chronically ill patients by comparing patient groups and investigating determinants.

The QAPICHE study was approved by the Ethical Committee of the Ghent University Hospital, Belgium (reference no. B670201939629) and is registered on ClinicalTrials.gov (identification no. NCT03925805). Written informed consent was obtained from all participants in the study.

### Study population

A total of 544 chronically ill people, recruited from June 2019 through June 2021, participated in the study. Of these participants, 287 (52.8%) were recruited via 56 general practitioners and the remaining 257 (47.2%) persons were recruited via 18 officially recognized Flemish patient organizations. Inclusion criteria were being an adult (aged ≥18 y) and being diagnosed with at least 1 of the following chronic diseases: any cardiometabolic disorder, any mental disorder, or any musculoskeletal disorder. These 3 disease groups were selected because they are associated with lower HRQOL than other disease groups and because musculoskeletal and cardiometabolic disorders are highly prevalent in Belgium (19). Patients with insufficient understanding of the Dutch language to complete the questionnaire were excluded.

### Measures

#### Health-related quality of life

The EuroQol-5 Dimension-5 Level questionnaire (EQ-5D-5L) is composed of a descriptive system covering 5 dimensions (ie, mobility, self-care, usual activities, pain/discomfort, and anxiety/depression) defined by 5 severity levels (ie, no problems, slight problems, moderate problems, severe problems, and extreme problems/unable to) from which a single index value or utility value can be calculated, ranging from 0 (death) to 1 (perfect health) and with negative values indicating health states perceived to be worse than death (20). Recently, an EQ-5D-5L value set was developed according to the health state preferences of the general population of Belgium (21). Possible index values of this value set range from −0.532 to 1. The EuroQol visual analogue scale (EQ-VAS) measures respondents’ self-rated health on a 0 (worst imaginable health) to 100 (best imaginable health) scale. However, focusing only on utility values would result in a loss of potentially relevant information. As such, analyzing the individual dimensions as categorical outcome (no problems vs any problems) is recommended (22).

The 12-Item Short Form Health Survey (SF-12) is a shortened version of the 36-Item Short Form Health Survey (SF-36) (23). The instrument contains 12 items that evaluate 8 domains pertaining to HRQOL: physical functioning, role-physical, bodily pain, general health, vitality, social functioning, role-emotional, and mental health. Both a physical component summary (PCS) score and a mental health component summary (MCS) score can be calculated according to a US general population scoring algorithm (24). The scores range from 0 to 100, with 0 representing the lowest level of health and 100 the highest level of health.

#### Psychological distress

The 14-item Hospital Anxiety and Depression Scale (HADS) (25) is a brief instrument to determine the presence of anxiety and depressive states. Each item has a 4-point response scale (range, 0–3); 7 items are related to anxiety and 7 items to depression. Item scores can be added to obtain a score for the anxiety subscale (HADS-anxiety) and the depression subscale (HADS-depression) separately. A maximum score of 21 can be achieved per subscale. The higher the overall score, the higher the levels of anxiety and depression.

#### Polypharmacy

Medication use was assessed with the question, “Which medication are you currently taking?” Medicines from the same pharmacologic subgroup (eg, fast-acting and long-acting insulin) were considered separate medicines. Although no consensus exists on the definition of polypharmacy, the QAPICHE study defined polypharmacy as the use of 5 or more medications concurrently (10). This value is linked to adverse outcomes such as disability, frailty, and mortality (26,27). For informational purposes, we added 2 more categories (5 to <10 medications and ≥10 medications).

#### Sociodemographic characteristics

Patient demographic variables included age (18–44, 45–54, 55–64, ≥65 y), sex (male, female), marital status (married, widowed/divorced, single), and educational attainment. Educational attainment was classified into low (lower secondary education or less), intermediate (higher secondary education), and high (higher education), according to the International Standard Classification of Education (28).

#### Multimorbidity

Participants were asked to respond yes or no to having a chronic condition from a list of 24 chronic conditions. A response of yes indicated a condition that had been confirmed by a physician or for which the participant was taking prescribed drugs. The following chronic conditions and diseases were measured: cardiovascular disease, stroke, heart failure (including valve problems or replacement), hypertension, high cholesterol, chronic joint disease (eg, rheumatoid arthritis), chronic musculoskeletal conditions causing pain or limitation, osteoporosis, chronic disease of the back/neck, any cancer in the previous 5 years, chronic lung disease (eg, asthma, chronic obstructive pulmonary disease), type 1 and 2 diabetes, thyroid disorder, chronic kidney disease, chronic urinary problems (eg, incontinence), chronic stomach problems, colon problems (eg, irritable bowel, Crohn disease), chronic hepatitis, headache disorder (eg, migraine), depression/chronic anxiety, mental disorder (eg, schizophrenia), neurologic disorder (eg, epilepsy), Alzheimer disease/dementia, and chronic skin disease. Defining multimorbidity as the presence of 2 or more chronic diseases is not universally accepted, especially when highly prevalent conditions (eg, hypertension, osteoporosis) are included, because these result in higher prevalence rates of multimorbidity (29). Hence, we defined multimorbidity as having 3 or more concurrent diseases and used the simple count method (30).

### Statistical analysis

First, we performed descriptive analyses. For continuous variables, we calculated means and SDs; for categorical variables, we calculated percentages. After checking the assumptions, multiple linear regression models assessed the association between polypharmacy and HRQOL. Likewise, we used logistic regression to investigate the association between polypharmacy and each EQ-5D-5L dimension. Initially, in both analyses, we adjusted the model for age, sex, and educational attainment to account for potential confounding; we then applied a further adjustment for multimorbidity. We used SPSS statistical software version 27.0 (IBM Corp) to analyze data. Significance levels were set at *P* < .05.

## Results

The mean (SD) age of patients was 58.6 (15.8) years, and 67.1% were women (Table 1). Low and high education was reported by 28.4% and 39.1% of respondents, respectively. Multimorbidity occurred in 63.4% of the participants. The mean number of medications consumed by patients was 5.7 (SD, 3.8; range 0–20), and most reported polypharmacy (54.9%). Of 528 patients, 45.1% (n = 238) used 0 to <5 medications per day, 42.6% (n = 225) used 5 to <10 medications per day, and 12.3% (n = 65) used 10 or more medications per day. The prevalence of polypharmacy was higher among patients with multimorbidity than among patients without multimorbidity (67.3% vs 33.7%, *P* < .001).

**Table 1:** Characteristics of Participants in the QAPICHE Study, by Polypharmacy Status, Flanders, Belgium, June 2019–June 2021^a^

Characteristic	Overall (N = 544)^b^	0 to <5 Medications (n = 238)	5 to <10 Medications (n = 225)	≥10 Medications (n = 65)
**Age, mean (SD), y**	58.6 (15.8)	53.6 (16.4)	61.4 (14.4)	64.0 (12.9)
**Age group, y**				
18–44	19.3 (105/543)	69.2 (72/104)	27.9 (29/104)	2.9 (3/104)
45–54	18.2 (99/543)	44.9 (44/98)	39.8 (39/98)	15.3 (15/98)
55–64	22.8 (124/543)	39.8 (49/123)	45.5 (56/123)	14.6 (18/123)
≥65	39.6 (215/543)	35.6 (72/202)	50.0 (101/202)	14.4 (29/202)
**Sex**				
Female	67.1 (365/544)	46.9 (167/356)	41.3 (147/356)	11.8 (42/356)
Male	32.9 (179/544)	41.3 (71/172)	45.3 (78/172)	13.4 (23/172)
**Educational attainment^c^**				
Low	28.4 (154/542)	40.1 (59/147)	45.6 (67/147)	14.3 (21/147)
Intermediate	32.5 (176/542)	39.7 (69/174)	46.0 (80/174)	14.4 (25/174)
High	39.1 (212/542)	52.9 (109/206)	37.9 (78/206)	9.2 (19/206)
**Employment status**				
Employed	20.1 (109/541)	62.0 (67/108)	35.2 (38/108)	2.8 (3/108)
Unemployed	5.7 (31/541)	53.3 (16/30)	40.0 (12/30)	6.7 (2/30)
Student	1.3 (7/541)	85.7 (6/7)	14.3 (1/7)	0
Disability	26.4 (143/541)	44.4 (63/142)	38.7 (55/142)	16.9 (24/142)
Pensioner	44.9 (243/541)	35.7 (82/230)	49.6 (114/230)	14.8 (34/230)
Other	1.5 (8/541)	37.5 (3/8)	37.5 (3/8)	25.0 (2/8)
**Marital status**				
Single	18.4 (100/543)	52.0 (52/100)	40.0 (40/100)	8.0 (8/100)
Married or cohabiting	66.5 (361/543)	45.0 (159/353)	42.8 (151/353)	12.2 (43/353)
Divorced	8.7 (47/543)	37.2 (16/43)	46.5 (20/43)	16.3 (7/43)
Widow(er)	6.4 (35/543)	34.4 (11/32)	43.8 (14/32)	21.9 (7/32)
**Disease type**				
Cardiovascular disease	58.8 (317/539)	34.2 (106/310)	50.6 (157/310)	15.2 (47/310)
Mental disease	27.1 (146/539)	42.4 (61/144)	43.8 (63/144)	13.9 (20/144)
Musculoskeletal disease	70.1 (378/539)	43.1 (160/371)	42.6 (158/371)	14.3 (53/371)
Diabetes	27.1 (146/539)	33.1 (47/142)	50.7 (72/142)	16.2 (23/142)
Other	70.3 (379/539)	38.3 (142/371)	46.4 (172/371)	15.4 (57/371)
**Multimorbidity^d^**				
No	36.6 (197/538)	66.3 (126/190)	30.5 (58/190)	3.2 (6/190)
Yes	63.4 (341/538)	32.7 (110/336)	49.7 (167/336)	17.6 (59/336)

a Data were from a cross-sectional study (the QAPICHE study, an acronym for “quality of life in patients with chronic disease”) conducted in Flanders, Belgium (18). Values are percentage (number/total) unless otherwise indicated.

b 544 Adults responded to the EuroQol-5 Dimension-5 Level (EuroQol-5D-5L) questionnaire. Denominators may be <544 because of missing data. Likewise, across rows, the denominator for each category of medication use may not add to numerator in first column because of missing data. Column percentages may not add to 100 because of rounding.

c Classified into low (lower secondary education or less), intermediate (higher secondary education), and high (higher education), according to the International Standard Classification of Education (28).

d Defined as ≥3 concurrent diseases.

### HRQOL/psychological distress outcomes

**Figure 1:**
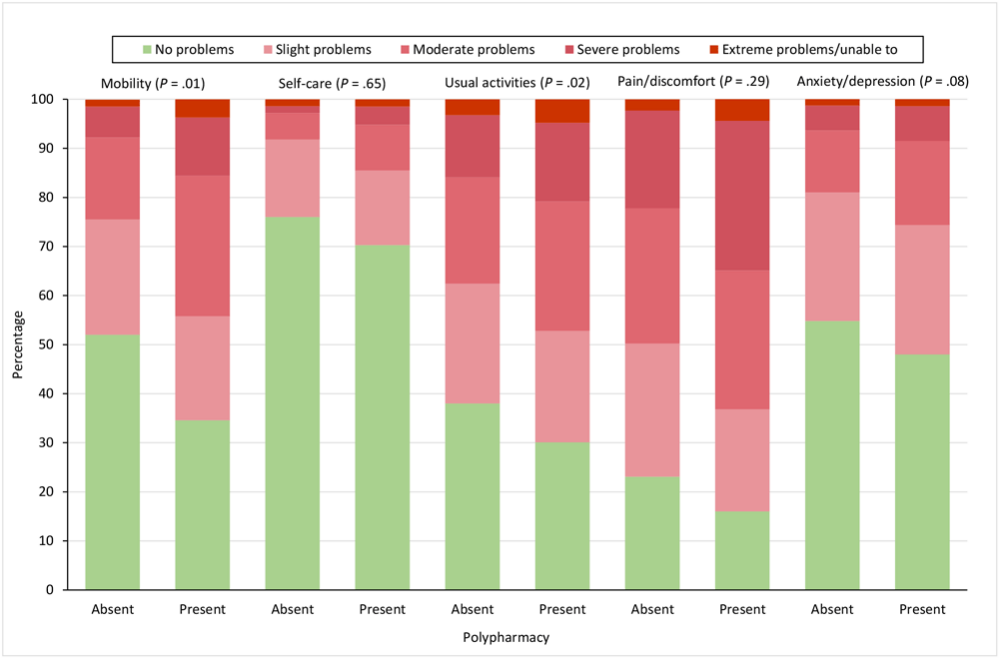
Percentage of patients reporting problems on the 5 EQ-5D-5L dimensions, by whether patient reported polypharmacy, defined in study as use of ≥5 medications. *P *values were determined by logistic regression and were adjusted for age, sex, educational attainment, and multimorbidity; significance level set at <.05. Abbreviation: EQ-5D-5L, EuroQol-5 Dimension-5 Level questionnaire.

Patients with polypharmacy had a higher frequency of problems on all EQ-5D-5L dimensions compared with patients without polypharmacy (Figure 1). We found significant differences for the dimensions of mobility (*P* = .01) and usual activities (*P* = .02). In general, compared with patients without polypharmacy, patients with polypharmacy reported worse EQ-5D-5L index values (0.69 vs 0.60; *P* < .001), EQ-VAS scores (62.6 vs 58.0; *P* = .005), SF-12 PCS scores (39.2 vs 35.0; *P* < .001), SF-12 MCS scores (43.3 vs 42.8; *P* = .68), HADS-anxiety scores (7.5 vs 7.6; *P* = .88), and HADS-depression scores (6.5 vs 7.3; *P* = .06).

**Table:** 

Level of problems	Mobility (*P* = .01)		Self-care (*P* = .65)		Usual activities (*P* = .02)		Pain/discomfort (*P* = .29)		Anxiety/depression (*P* = .08)	
	No polypharmacy	Polypharmacy	No polypharmacy	Polypharmacy	No polypharmacy	Polypharmacy	No polypharmacy	Polypharmacy	No polypharmacy	Polypharmacy
No problems	52.0	34.6	76.0	70.3	38.0	30.1	23.1	16.0	54.8	48.0
Slight problems	23.5	21.2	15.8	15.2	24.4	22.7	27.1	20.8	26.2	26.4
Moderate problems	16.7	28.6	5.4	9.3	21.7	26.4	27.6	28.3	12.7	17.1
Severe problems	6.3	11.9	1.4	3.7	12.7	16.0	19.9	30.5	5.0	7.1
Extreme problems/unable to	1.4	3.7	1.4	1.5	3.2	4.8	2.3	4.5	1.4	1.5

**Figure 2:**
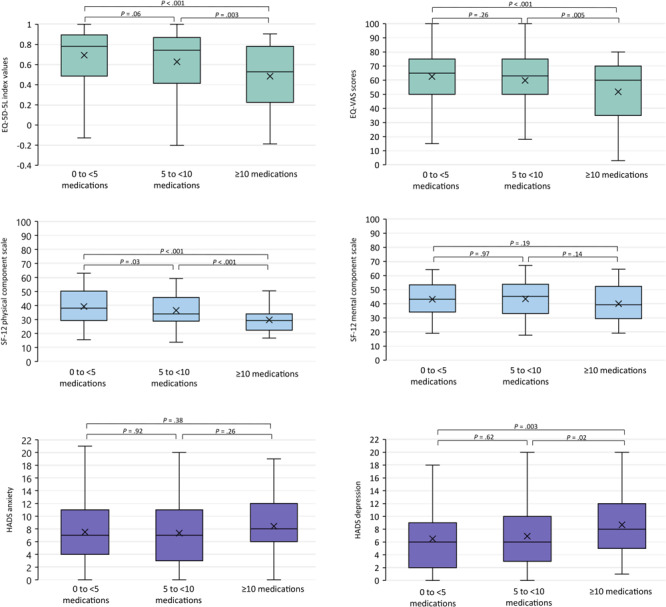
EQ-5D-5L index, EQ-VAS, SF-12 physical component score, SF-12 mental health component score, HADS anxiety subscale, and HADS depression subscale, by medication use. The horizontal bar inside the boxes indicates the median, the x indicates the mean, and the lower and upper ends of the boxes are the first and third quartiles. The whiskers indicate minimum and maximum values. *P *values were determined by *F* test (1-way analysis of variance) and adjusted for age, sex, educational attainment, and multimorbidity; significance level set at <.05. Abbreviations: EQ-5D-5L, EuroQol 5 Dimension-5 Level questionnaire; EQ-VAS, EuroQol visual analogue scale; HADS, Hospital Anxiety and Depression Scale; SF-12, 12-Item Short Form Health Survey.

In the analysis of HRQOL/psychological distress according to medication use, divided into 3 groups (0 to <5 medications, 5 to <10 medications, ≥10 medications), the higher the medication use, the worse the results on the EQ-5D-5L, the SF-12, and both HADS subscales (Figure 2).

**Table:** 

Item	0 to <5 medications			5 to <10 medications			≥10 medications		
	Mean (SD)	Median (interquartile range)	Minimum–maximum	Mean (SD)	Median (interquartile range)	Minimum–maximum	Mean (SD)	Median (interquartile range)	Minimum–maximum
EQ-5D-5L index score	0.7 (0.3)	0.8 (0.5 to 0.9)	−0.1 to 1	0.6 (0.3)	0.7 (0.4 to 0.9)	−0.2 to 1	0.5 (0.3)	0.5 (0.2 to 0.8)	−0.2 to 0.9
EQ-VAS	62.6 (18.2)	65.0 (50.0 to 75.0)	15 to 100	59.9 (17.6)	63.0 (50.0 to 75.0)	18 to 100	51.7 (20.0)	60.0 (35.0 to 70.0)	3 to 80
SF-12 PCS	39.2 (11.9)	38.1 (29.3 to 50.2)	16 to 63	36.4 (11.0)	34.0 (28.8 to 45.7)	14 to 59	29.7 (8.7)	29.3 (22.3 to 34.0)	17 to 50
SF-12 MCS	43.3 (11.4)	43.3 (34.2 to 53.4)	19 to 64	43.5 (12.3)	45.3 (33.1 to 53.9)	18 to 67	40.1 (13.1)	39.3 (29.4 to 52.3)	19 to 64
HADS-anxiety	7.5 (5.0)	7.0 (4.0 to 11.0)	0 to 21	7.3 (4.9)	7.0 (3.0 to 11.0)	0 to 20	8.4 (4.3)	8.0 (6.0 to 12.0)	0 to 19
HADS-depression	6.5 (4.8)	6.0 (2.0 to 9.0)	0 to 18	6.9 (4.8)	6.0 (3.0 to 10.0)	0 to 20	8.7 (4.6)	8.0 (5.0 to 12.0)	1 to 20

### Associations between polypharmacy and HRQOL/psychological distress

In the fully adjusted regression model (age, sex, educational attainment, multimorbidity), polypharmacy was significantly associated with lower HRQOL only in terms of EQ-5D-5L index (β = −0.12; *P* = .008), EQ-VAS (β = −0.11; *P* = .01), and SF-12 PCS (β = −0.15; *P* = .002) (Table 2). We observed no significant associations between polypharmacy and psychological distress (HADS) nor with the SF-12 MCS in the fully adjusted model.

**Table 2:** Associations Between Polypharmacy and Health-Related Quality of Life/Psychological Distress Among Participants in the QAPICHE Study, Flanders, Belgium, June 2019–June 2021^a^

Measure	Unadjusted model	Model 1^b^	Model 2^c^
	β (*P* value)	β (*P* value)	β (*P* value)
EQ-5D-5L index	−0.16 (<.001)	−0.21 (<.001)	−0.12 (.008)
EQ-VAS	−0.12 (.005)	−0.19 (<.001)	−0.11 (.01)
SF-12 physical component score (PCS)	−0.18 (<.001)	−0.21 (<.001)	−0.15 (.002)
SF-12 mental component score (MCS)	−0.02 (.68)	−0.11 (.01)	−0.01 (.80)
HADS anxiety subscale	0.01 (.88)	0.09 (.03)	0 (.99)
HADS depression subscale	0.08 (.06)	0.13 (.003)	0.03 (.57)

Abbreviations: EQ-5D-5L, EuroQol-5 Dimension-5 Level questionnaire; EQ-VAS, EuroQol visual analogue scale; HADS, Hospital Anxiety and Depression Scale; SF-12, 12-Item Short Form Health Survey.

a Determined by linear regression; *P* < .05 considered significant.

b Model 1 adjusted for age, sex, and educational attainment.

c Model 2 adjusted for age, sex, educational attainment, and multimorbidity.

## Discussion

Literature suggests that patients with polypharmacy have impaired HRQOL outcomes (13,14). This study examined the relationship between polypharmacy and HRQOL among adults with diverse chronic diseases, accounting for comorbidity. Most studies that investigated the association between polypharmacy and HRQOL focused on single diseases and used only 1 instrument. Our study, however, focused on several types of chronic diseases and used several instruments: HRQOL was measured via the EQ-5D-5L and the SF-12, and psychological distress was measured via the HADS. Information on the different aspects of HRQOL is important for aligning medical treatment to patient needs.

We found that most chronically ill patients reported polypharmacy, and as expected, the prevalence of polypharmacy was higher among patients with multimorbidity, in line with previous findings (31,32). The management of multiple chronic conditions often requires multiple prescriptions, increasing the risk of polypharmacy in patients with multimorbidity. Polypharmacy might affect a patient’s well-being. We found that patients with high levels of medication use had lower HRQOL and higher levels of psychological distress compared with patients with low levels of medication use. However, differences were clinically relevant only for the EQ-5D-5L index (33) and the SF-12 PCS (34). After adjusting for covariates (age, sex, educational attainment, and multimorbidity), results from the regression analysis showed that polypharmacy was negatively associated with the EQ-5D-5L index, the EQ-VAS, and the SF-12 PCS. No significant associations were found for the mental component of HRQOL nor psychological distress, suggesting an unfavorable effect of polypharmacy only on the physical domain of patients’ HRQOL. Indeed, overprescription of medication can cause increased risk of adverse drug–drug or drug–disease interactions, ultimately affecting physical functioning (35). However, inconsistency remains; some studies showed no significant association of polypharmacy with SF-12 MCS scores (16), while others found polypharmacy to be linked to an increased risk of depression, because some medications tend to cause or worsen depressive symptoms (36,37).

Pharmacologic treatment can be modified; hence, targeted interventions are recommended. Further research should investigate which medications affect the physical aspects of HRQOL. Medications that may cause harmful drug–drug or drug–disease interactions can be reduced or eliminated, while the remaining medications can focus on the patient’s most important functional priorities (38). Moreover, our results support the need for health care professionals to be aware of the negative effects of polypharmacy on HRQOL and to recognize drug-related adverse events, especially when treating patients with multimorbidity. In addition, health care professionals should assess the potential benefits and harms of prescribing multiple drugs to achieve ideal pharmacotherapy and to prescribe the medicines that can improve the HRQOL of this group (17). Evidence-based guidelines for patients with multimorbidity can help to inform deprescribing decisions, defined as “the process of withdrawal of an inappropriate medication, supervised by a health care professional with the goal of managing polypharmacy and improving outcomes” (39).

### Limitations and strengths

This study has several limitations. First, the study had a cross-sectional design; therefore, no statements about causality can be made. Second, bias toward more favorable results may have occurred because of selective nonresponse effects: patients with a more severe disease profile may not have completed the questionnaire (40,41). Third, the outbreak of the COVID-19 pandemic occurred during the data collection process. As such, pandemic-related stress may have worsened the HRQOL of patients who were included in the study during the pandemic. Fourth, data were self-reported, which might have produced recall bias. Fifth, because of ethical and privacy restrictions, we did not have access to patients’ medical records to evaluate medication use.

This study also had important strengths. First is the use of 3 international, validated instruments to assess HRQOL and psychological distress (ie, the EQ-5D-5L, the SF-12, and HADS). Second, we selected several variables to control for confounding in the regression analysis, including multimorbidity. However, medication use and multimorbidity are strongly correlated; hence, polypharmacy is commonly used as a proxy for multimorbidity. As a result, multimorbidity — not polypharmacy — may be the underlying cause of lower HRQOL (38,42). Nonetheless, polypharmacy remained significantly associated with HRQOL, even after adjustment for multimorbidity, indicating that polypharmacy independently affects HRQOL (17). Additionally, information on disease duration and disease severity was not available and not controlled for in our analyses, although they can affect both polypharmacy and HRQOL.

### Conclusion

In line with previous evidence, our study found that polypharmacy was negatively associated with the physical domain of HRQOL, but not the mental domain, among patients with chronic diseases. These results may be especially important for patients with multimorbidity, given their greater risk of polypharmacy.
